# Species-specific modulation of nitro-oxidative stress and root growth in monocots by silica nanoparticle pretreatment under copper oxide nanoparticle stress

**DOI:** 10.1186/s12870-025-06193-7

**Published:** 2025-02-13

**Authors:** Kamilla Kovács, Ádám Szierer, Enikő Mészáros, Árpád Molnár, Andrea Rónavári, Zoltán Kónya, Gábor Feigl

**Affiliations:** 1 Department of Plant Biology, University of Szeged, H-6726 Szeged, Közép fasor 52, Szeged, Hungary; 2 Doctoral School of Biology, University of Szeged, Szeged, Hungary; 3 Department of Applied and Environmental Chemistry, University of Szeged, Szeged, Hungary

**Keywords:** Monocotyledons, Copper oxide and silica nanoparticles, Root growth inhibition, Nitro-oxidative stress response, Species-specific response

## Abstract

**Background:**

Abiotic stressors such as heavy metals and nanoparticles pose significant challenges to sustainable agriculture, with copper oxide nanoparticles (CuO NPs) known to inhibit root growth and induce oxidative stress in plants. While silica nanoparticles (SiO_2_ NPs) have been shown to increase abiotic stress tolerance, their role in mitigating CuO NP-induced stress in crops, especially monocots, remains poorly understood. This study addresses this critical knowledge gap by investigating how SiO_2_ NP pretreatment modulates CuO NP-induced stress responses, with a particular focus on root growth inhibition and nitro-oxidative stress pathways.

**Results:**

Using an in vitro semihydroponic system, seeds were pretreated with varying concentrations of SiO_2_ NPs (100–800 mg/L) before exposure to CuO NPs at levels known to inhibit root growth by 50%. SiO_2_ NP pretreatment alleviated CuO NP-induced root growth inhibition in sorghum, wheat, and rye but intensified it in triticale. These responses are associated with species-specific alterations in reactive signaling molecules, including a reduction in nitric oxide levels and an increase in hydrogen sulfide in sorghum, a decrease in superoxide anion levels in rye, and elevated hydrogen peroxide levels in wheat. Protein tyrosine nitration, a marker of nitro-oxidative stress, was reduced in most cases, further indicating the stress-mitigating role of SiO_2_ NPs. These signaling molecules were selected for their established roles in mediating oxidative and nitrosative stress responses under abiotic stress conditions.

**Conclusions:**

SiO_2_ NP pretreatment modulates CuO NP-induced stress responses through species-specific regulation of reactive oxygen and nitrogen species, demonstrating its potential as a tool for enhancing crop resilience. These findings advance the understanding of nanoparticle‒plant interactions and provide a foundation for future applications of nanotechnology in sustainable agriculture.

**Clinical trial number:**

Not applicable.

**Supplementary Information:**

The online version contains supplementary material available at 10.1186/s12870-025-06193-7.

## Highlights

SiO_2_ NP pretreatment counteracts CuO NP-induced stress in sorghum, wheat, and rye roots.

Species-specific effects on the ROS and RNS levels under the SiO_2_ and CuO NP treatments.

CuO NP stress increases protein nitration, which is reduced by SiO_2_ NPs in most cases.

NPs impact plant stress pathways, revealing complex mitigation mechanisms.

**Supplementary Information:**

The online version contains supplementary material available at 10.1186/s12870-025-06193-7.

## Introduction

The increasing prevalence of nanotechnology has led to the growing presence of nanoparticles (NPs) across a range of sectors, including agriculture. NPs are used in fertilizers, pest control and seed treatment [1–5], and their growing presence in agricultural soils raises concerns regarding their potential impacts on crop growth and soil ecosystems. These particles enter agricultural soils in two ways: directly through their application and indirectly via other human activities [1, 6–8]. Once present in the soil, they persist and may alter the physical and chemical properties of the soil, affect plant growth, and impact soil microbial communities [9]. Furthermore, NPs are often present in sewage sludge, and their application as fertilizers may result in further contamination of soils and interactions with crops [10].

The uptake, transport, and accumulation of NPs in plants are influenced by particle characteristics, including size, shape, and composition, as well as by plant anatomy [11]. Typically, plant roots absorb NPs through various membrane structures, including transporters, aquaporins, carrier proteins, and channels. However, NPs can also enter through aerial tissues, such as the cuticle, stomata, and trichomes (12–13). Once inside a plant, NPs initially travel through the apoplast but may encounter barriers at the cell membrane or Casparian strip, necessitating a switch to the symplastic pathway [13]. Additionally, larger NP clusters can impede cell wall penetration [14]. Following absorption, NPs may induce cellular stress by releasing metal ions or generating reactive oxygen and nitrogen species (ROS and RNS), which can damage cell components, inhibit proteins, and destabilize lipids and DNA [15–17].

Copper oxide (CuO) nanoparticles are of particular interest because of the essential role of copper as a micronutrient in both plant and human metabolism. CuO NPs are employed in a multitude of applications, including fertilizers, electronics and industrial products, thereby increasing the likelihood of environmental exposure [9, 18]. Moreover, CuO NPs are widely employed because of their dual role as micronutrient suppliers and antimicrobial agents (19–20). However, their accumulation in soils has been associated with phytotoxic effects, such as oxidative stress, root growth inhibition, and disruptions in plant physiological processes [21]. These effects challenge the development of sustainable agricultural practices. The available evidence on CuO NP exposure in monocots indicates that the plant responses observed are variable. For example, rice (*Oryza sativa* L.) typically inhibits growth, whereas wheat (*Triticum aestivum* L.) responses vary with CuO NP concentration and exposure conditions. Some studies have even noted positive growth effects [21–23]. Corn (*Zea mays* L.) frequently demonstrates reduced growth in both hydroponic and soil-based environments, whereas barley (*Hordeum vulgare* L.) shows responses that depend on the NP concentration and exposure duration [21, 24–26]. The growth of aquatic monocots, including *Landoltia punctata* and *Lemna minor*, is consistently inhibited by CuO NPs, irrespective of their concentration or size [27–30].

CuO NPs induce oxidative stress in plants, primarily as a consequence of the release of soluble copper ions, which disrupts cellular homeostasis and results in elevated ROS levels. This oxidative stress can result in the peroxidation of lipids, denaturation of proteins, and ultimately, cell death [31–33].

Silicon (Si), the second most abundant element in the Earth’s crust, varies in its soil content (23–35 wt%) but remains largely insoluble (34–35). The uptake of silicon by plants, although not considered an essential nutrient, depends on species-specific differences in Si transporter activity (36–37). In addition to being vital for human health, Si plays a pivotal role in enhancing plant tolerance to abiotic stressors [38–40]. Research indicates that Si mitigates salt stress by stabilizing root membrane structures, preventing salt influx, alleviating drought stress through osmotic balance maintenance and mineral uptake support, and reducing heat stress by strengthening xylem cell walls [41–44]. Furthermore, Si has been demonstrated to alleviate heavy metal stress by forming complexes with metals, limiting their mobility within plants and promoting ion compartmentalization (45, 46). Additionally, Si influences plant morphology, promoting increased leaf area and root length and altering hormone production [47].

While recent research has highlighted the potential of nanoparticles such as silicon dioxide nanoparticles (SiO_2_ NPs or silica NPs) for mitigating abiotic stress, studies exploring their role in counteracting the specific phytotoxic effects of CuO NPs are rare. SiO_2_ NPs may enhance plant antioxidant defense mechanisms, thereby potentially mitigating the oxidative stress (48–49) caused by CuO NPs. SiO_2_ NPs have been demonstrated to stimulate controlled ROS production, thereby priming plant defenses against subsequent stressors such as CuO NPs [11]. The introduction of SiO_2_ NPs has been demonstrated to upregulate the expression of antioxidant enzymes, which play a pivotal role in the detoxification of the ROS generated during CuO NP exposure. Available evidence suggests that SiO_2_ NPs may increase plant resilience by modulating the expression of genes related to stress responses and improving the antioxidant defense system. For example, Abdelrhim et al. [50] reported that SiO_2_ NPs significantly increased the activities of antioxidant enzymes, including superoxide dismutase (SOD), ascorbate peroxidase (APX), catalase (CAT), and glutathione peroxidase (GPX), in wheat, thereby counteracting ROS and reducing lipid peroxidation. Similarly, Jurkow et al. [51] reported that SiO_2_ NP treatment of oakleaf lettuce seedlings resulted in elevated glutathione (GSH) levels, indicating increased antioxidant defense. Furthermore, Al-Mokadem et al. [52] reported that SiO_2_ NPs stimulate the activities of enzymes such as CAT, SOD, and peroxidase (POX), thereby increasing the ability of plants to manage stress.

Building on previous findings that demonstrated 50% inhibition of root growth caused by CuO NPs, which induced varying nitro-oxidative responses in the roots of relatively sensitive sorghum at 50 mg/L and tolerant wheat, rye, and triticale at 150 mg/L [53], this study explores, for the first time, the potential mitigation of CuO NP-induced stress through SiO_2_ NP priming in monocot seedlings. Preliminary experiments included treatments in which seeds were pretreated with SiO_2_ NPs alone without exposure to CuO NPs (data not shown). These controls showed that SiO_2_ NPs positively influenced root growth under nonstressed conditions (Fig. S1) but did not induce significant changes in the ROS and RNS levels. These findings suggest that the observed mitigation effects of SiO_2_ NPs are specific to their interaction with CuO NP-induced stress rather than being inherent to SiO_2_ NP treatment alone.

In light of the inhibitory effects of CuO NPs on plant growth and development, the utilization of SiO_2_ NPs represents a promising strategy for alleviating such damage [54]. Seed priming with NPs, also known as nanopriming, represents a novel approach to increase plant resilience against heavy metal (HM) stress [55]. This method leverages the distinctive characteristics of NPs, including their high surface area-to-volume ratio and reactivity, to facilitate their passage through seed coats and direct delivery of growth regulators and nutrients to the embryo [56]. Nanopriming has been demonstrated to regulate plant physiological and biochemical processes, resulting in increased seed germination, vigor, and stress tolerance [57]. Studies have demonstrated that nanopriming with metal oxide nanoparticles, such as zinc oxide (ZnO) and SiO_2_, can effectively mitigate the toxicity of HMs by reducing the accumulation of ROS, activating antioxidant enzymes (e.g., SOD, CAT, and POX), and enhancing nutrient uptake. For example, studies have demonstrated that ZnO NPs can mitigate the toxicity of cadmium in crops such as rice and tomato (58–59). Similarly, SiO_2_ NPs have been shown to reduce oxidative damage and heavy metal concentrations, thereby increasing plant health and productivity (60–61). Although nanopriming has demonstrated efficacy in enhancing seed germination, seedling growth, and stress tolerance in select plant species, it is imperative to address potential constraints and concerns, including toxicity, variability in effectiveness, and environmental impacts, to guarantee the secure and sustainable integration of this technology in agricultural practices [62].

However, existing research often lacks comparative analysis across different crop species, leaving a critical gap in understanding how species-specific factors influence the interaction between SiO_2_ and CuO NPs. Addressing this gap is essential for developing targeted strategies to increase crop resilience under nanoparticle-induced stress, which is a growing challenge in modern agriculture. Our study fills this void by investigating the potential of SiO_2_ NP pretreatment to mitigate CuO NP-induced stress in four agriculturally significant monocot species. By focusing on species-specific effects and elucidating changes in key signaling molecules and protein tyrosine nitration patterns, this research advances our understanding of nanoparticle interactions in plant stress physiology and provides actionable insights for sustainable agricultural practices.

In this study, we investigated the potential of SiO_2_ NP pretreatment to mitigate CuO NP-induced stress in four monocot species: sorghum, wheat, rye, and triticale. These species present a range of sensitivities to abiotic stress, providing a comparative framework for assessing the efficacy of SiO_2_ NPs in alleviating phytotoxic effects. Using a semihydroponic in vitro system, we focused on understanding how SiO_2_ NP pretreatment modulates early root development, the equilibrium of signaling molecules (reactive oxygen, nitrogen and sulfur species), and protein tyrosine nitration under CuO NP stress.

We hypothesized that SiO_2_ NP pretreatment can alleviate CuO NP-induced stress in a species-dependent manner by modulating nitro-oxidative signaling pathways. The primary objectives of this study were as follows:

1. Evaluation of the impact of SiO_2_ NP pretreatment on root growth inhibition caused by CuO NPs across different monocot species.
2. Changes in the levels of key signaling molecules and protein tyrosine nitration in response to SiO_2_ NP pretreatment under CuO NP stress were investigated.
3. The species-specific differences in the interactions between SiO_2_ and CuO NPs were explored to identify effective strategies for mitigating the adverse effects of CuO NPs on agricultural productivity.

## Materials and methods

### Plant materials

The seeds of the plant species used in the experiments were provided by Cereal Research Nonprofit Ltd., Szeged, Hungary. The monocot species used included sorghum (*Sorghum bicolor* L., GK Emese), rye (*Secale cereale* L., Wibro), triticale *(×Triticosecale*, GK Maros), and wheat (*Triticum aestivum* L., GK Békés). The selection of sorghum, wheat, rye, and triticale as monocot species for this study is rooted in their significant ecological and economic relevance, as well as their varying tolerances to abiotic stresses. Sorghum, the fifth most important cereal crop globally, is a C4 plant known for its water-use efficiency, diverse phenotypes, and ability to thrive under adverse conditions with minimal inputs [63]. They also exhibit tolerance to abiotic stresses such as salinity and alkalinity [64]. Wheat, a staple crop, provides a substantial portion of the global caloric intake and is pivotal to food security [65]. Rye is renowned for its adaptability to marginal environments and strong resilience against abiotic stresses, making it an important cereal crop for challenging climates [66]. Triticale, a hybrid of wheat and rye, combines the high yield potential of wheat with the stress tolerance of rye, offering a versatile solution for sustainable agriculture [67]. These species present a diverse range of stress tolerance levels and agricultural roles, providing an ideal framework for investigating species-specific responses to nanoparticle treatments.

### Nanoparticles

The experiment was conducted using two distinct types of nanoparticles (copper oxide (CuO) and silicon dioxide (SiO_2_)). In previous studies, the CuO nanoparticles were characterized and the concentrations that inhibited root growth by 50% in each plant species were determined [53]. The concentrations were 50 mg/L for the relatively sensitive sorghum and 150 mg/L for the relatively tolerant rye, triticale, and wheat. The CuO nanoparticles were synthesized via a modified precipitation method, as previously described by Phiwdang et al. [68] and Molnár et al. [69]. Transmission electron microscopy revealed that the particles were ellipsoidal and rod-shaped, forming loose aggregates with an average particle size of 48.2 ± 6.3 nm [53]. The zeta potential of the particles was found to be 25.4 mV.

For the purpose of seed pretreatment, a 10–20 nm amorphous silicon dioxide nanopowder (Sigma‒Aldrich, 637238, see characteristics in Fig. S2) was prepared in a series of suspensions (100, 200, 400, and 800 mg/L) through sonication for a period of 30 min. The seeds were immersed in the aforementioned suspensions for a period of six hours at room temperature in the absence of light. Following the completion of the treatment, the seeds were removed from the suspension, dried for a period of 24 h, and stored refrigerated until use.

The concentration ranges of SiO_2_ NPs were selected on the basis of preliminary experiments, which revealed a positive, concentration-dependent effect of SiO_2_ NP pretreatment alone on root growth across the four monocot species.

### Experimental setup

The experiments were conducted in vitro under semihydroponic conditions in sterile Petri dishes lined with two layers of filter paper. The filter papers were moistened with 5 mL of either deionized water (control) or a CuO nanoparticle suspension as previously described. Ten seeds were placed in each Petri dish and incubated in a controlled growth chamber for five days. The growth conditions included a 12-hour light/dark cycle at 250 µmol/m⁻² s⁻¹ white LED illumination (5700 K) with far-red light supplementation (PSI, Drásov, Czech Republic), 55–60% humidity, and a temperature of 25 ± 2 °C.

### Measurement of root growth parameters

At the end of the growth cycle, primary root lengths were measured (PR length; mm), and the numbers of lateral (LR; pieces/primary root) and fibrous roots (root number, RN; pieces/seedlings) were counted.

### Detection of reactive signaling molecules

The detection of superoxide anions was conducted using 10 µM dihydroethidium (DHE) in Tris-HCl buffer (10 mM, pH 7.4) [70]. Hydrogen peroxide was quantified via the fluorophore Amplex Red (10-acetyl-3,7-dihydroxyphenoxazine) at a concentration of 50 µM [71]. Nitric oxide (NO) detection was conducted by incubating the root tips in a solution of DAF-FM DA (4-amino-5-methylamino-2’,7’-difluorofluorescein diacetate) at a concentration of 10 µM [72]. For the detection of peroxynitrite, samples were incubated in a solution of 10 µM APF (aminophenyl fluorescein) [73]. The detection of hydrogen sulfide was conducted via the fluorescent dye WSP-1 (Washington State Probe 1), which was used at a concentration of 15 mM in a 20 µM HEPES-NaOH buffer solution at pH 7.5 [74].

### Examination of cell wall modifications

The presence of quercetin was detected through the utilization of DPBA (2-aminoethyl diphenylborinate) at a concentration of 0.25% (weight/volume) in distilled water with 0.005% Triton X-100 [75]. The visualization of callose was achieved through the use of 0.1% aniline blue dye (dissolved in 1 M glycine) [76].

### Microscopic analysis of staining techniques

The root tips were examined under a Zeiss Axiovert 200 M inverted microscope (Carl Zeiss, Jena, Germany), which was equipped with a high-resolution digital camera (Axiocam HR, HQ CCD, Carl Zeiss, Jena, Germany). A variety of filters with distinct excitation and emission wavelengths were employed. To detect the reactive nitrogen species, filter set 10 (excitation range: 450–490 nm, emission range: 515–565 nm) was used. For DHE, DPBA, and WSP-1, filter set 9 (excitation range: 450–490 nm, emission range: 515–∞ nm) was employed. Aniline blue was visualized with filter set 49 (excitation: 365 nm, emission: 445–50 nm), whereas Amplex Red was observed with filter set 20HE (excitation: 546–12 nm, emission: 607–80 nm).

The images of the root division zone were analyzed via AxioVision Rel. 4.8 Software. The pixel intensity, which is proportional to the amount of detected molecules, was measured within a radius of 50 micrometres.

### Western blot analysis

Western blotting was performed to detect protein tyrosine nitration. The root samples were homogenized in a chilled mortar with extraction buffer (50 mM Tris-HCl, 0.1 mM EDTA, 0.1% Triton X-100, and 10% glycerol). The samples were subsequently centrifuged at 12,000 rpm for 20 min at 4 °C, after which the supernatants were denatured with nonreducing sample buffer. Denatured protein samples were loaded onto SDS‒polyacrylamide gels (4% stacking, 12% resolving) for electrophoresis. The separated proteins were transferred to PVDF membranes and incubated overnight at 4 °C with anti-3-nitrotyrosine antibodies at a dilution of 1:2000 [77]. A secondary antibody conjugated with alkaline phosphatase was used for immunodetection at a dilution of 1:10,000. The presence of nitrated proteins was confirmed through the use of the BCPIP/NBT reaction.

### Statistical analysis

The results are presented as the mean ± standard error (SE). Statistical analyses were performed via SigmaStat 12 software. One-way analysis of variance (ANOVA) was conducted to identify significant differences among the treatment groups. Duncan’s multiple range test (*P* ≤ 0.05) was applied post hoc to compare means and determine statistically significant differences within groups. For visual representation, the data are presented as the means ± standard errors (SEs), with distinct letters indicating significant differences between treatments.

## Results and discussion

### Effect of SiO_2_ NP seed pretreatment on the root growth of the tested species under CuO NP treatment with 50% growth inhibition

**Fig. 1:**
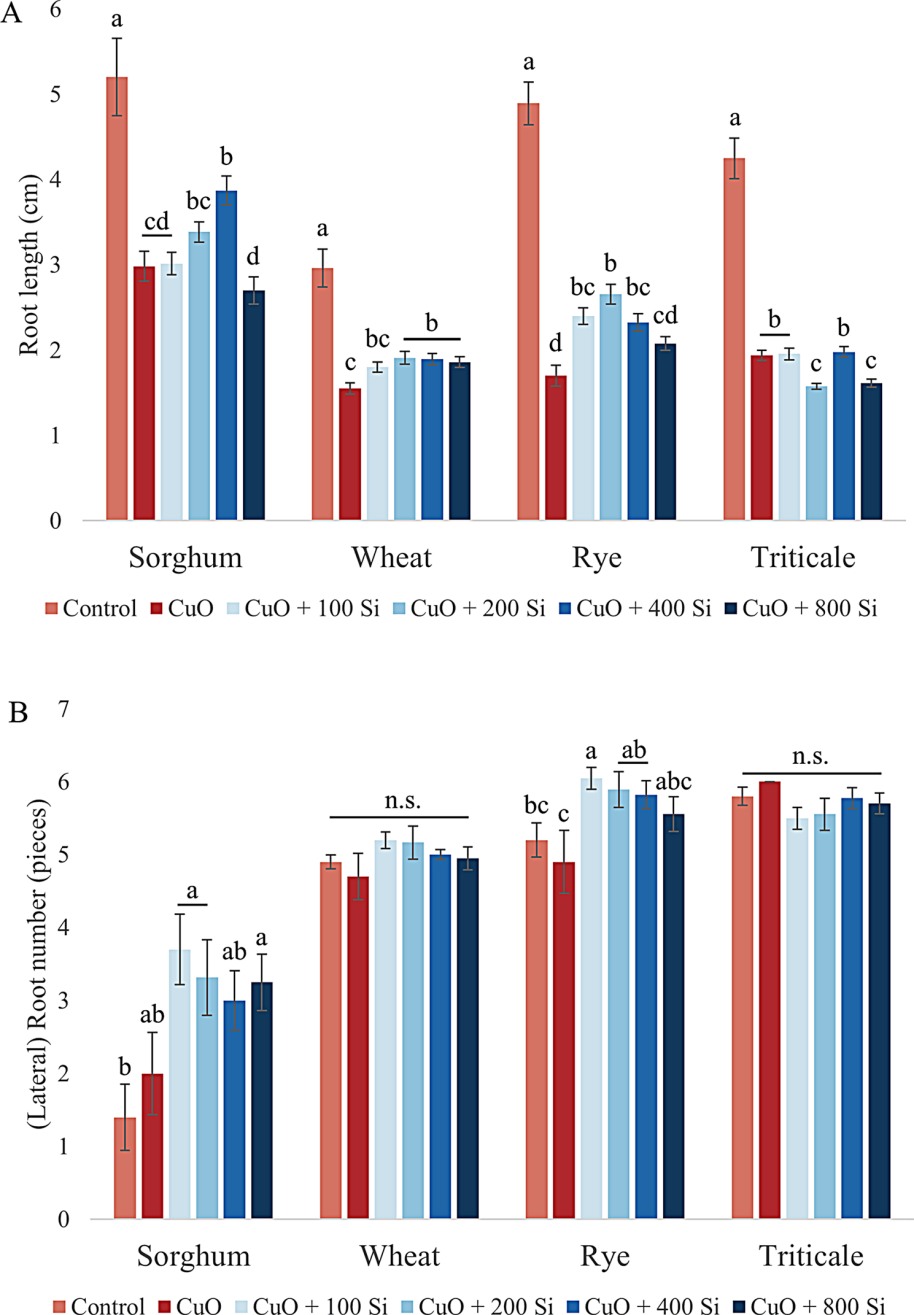
Effects of CuO nanoparticle stress and SiO_2_ nanoparticle pretreatment on the change in root length (**A**) and (lateral) root number (**B**) of sorghum, wheat, rye and triticale. The results are presented as the means ± standard errors (different letters indicate significant differences according to Duncan’s test; *P* ≤ 0.05; n.s., no significant change)

The data on root length indicate that the pretreatment of seeds with SiO_2_ NPs was able to counteract the inhibitory effect of CuO NPs on root length growth in sorghum, wheat and rye. However, at both tested concentrations, it also inhibited root growth in triticale (Fig. 1A).

The most significant positive outcome was observed in sorghum at the 400 mg/L SiO_2_ NP concentration (*P* = 0.017). In contrast, 800 mg/L pretreatment had a more pronounced inhibitory effect on main root growth compared to plants submitted to CuO NP stress alone. In the case of wheat, the application of a wider range of SiO_2_ NP pretreatments was observed to reduce CuO-induced root growth inhibition. The most significant reduction was achieved with concentrations of 200, 400 and 800 mg/L, which resulted in root lengths that were nearly equal, with the 400 mg/L concentration demonstrating the most favorable outcome (*P* = 0.027). In the case of rye, the application of SiO_2_ NP pretreatment at concentrations of 100, 200 and 400 mg/L resulted in a significant increase in root growth (*P* < 0,001), whereas the highest concentration resulted in a more modest but still discernible improvement in root growth compared with that of plants treated with only CuO NPs. The root growth inhibition observed in triticale was not attenuated by the application of SiO_2_ NPs at concentrations of 100 and 400 mg/L, which yielded results comparable to those observed in plants treated with CuO NPs alone. However, the application of 200 and 800 mg/L SiO_2_ NPs resulted in a significant further reduction in root length (*P* = 0.015 and 0.02, respectively).

Other parameters that provide insight into root development include root number (wheat, rye, triticale) and lateral root number (sorghum) (Fig. 1B). These results demonstrate that SiO_2_ NP pretreatment has a beneficial effect on the number of (lateral) roots of sorghum and rye plants. In contrast, neither CuO nor SiO_2_ NP treatment resulted in a significant change in root number in wheat and triticale. In sorghum, the CuO NP treatment resulted in a slight increase in the number of lateral roots. However, pretreatment with varying concentrations of SiO_2_ NPs led to a significant further increase in the number of lateral roots under CuO NP stress. The response observed in wheat was positive but not statistically significant. In the case of rye, the most significantly positive outcome was observed following a pretreatment of 100 mg/L (*P* = 0.007), whereas for higher concentrations, a continuous decrease was noted. Nevertheless, even in these instances, the root number remained greater than that observed in the seedlings cultivated on CuO NPs alone. Ultimately, the application of CuO NPs to triticale resulted in a slight increase in root number. However, the addition of silicon led to a slight decrease in root number.

Research has demonstrated that silicon is a vital component in the alleviation of stress processes in plants. Soltani et al. [78] reported that the exogenous application of silicon enhances growth and nutrient uptake in tubers of *Solanum tuberosum* (potato) plants. Furthermore, under conditions of cadmium stress, treatment with silicon nanoparticles resulted in a significant reduction in growth inhibition in moso bamboo [79]. Furthermore, the application of silicon increased the growth parameters of seed bean in soil contaminated with heavy metals and salts [80]. In accordance with the literature data, a preliminary series of experiments demonstrated that the effect of SiO_2_ NP pretreatment on the root growth of the four species was positive (data not shown).

### Examination of the equilibrium of reactive molecules

**Fig. 2:**
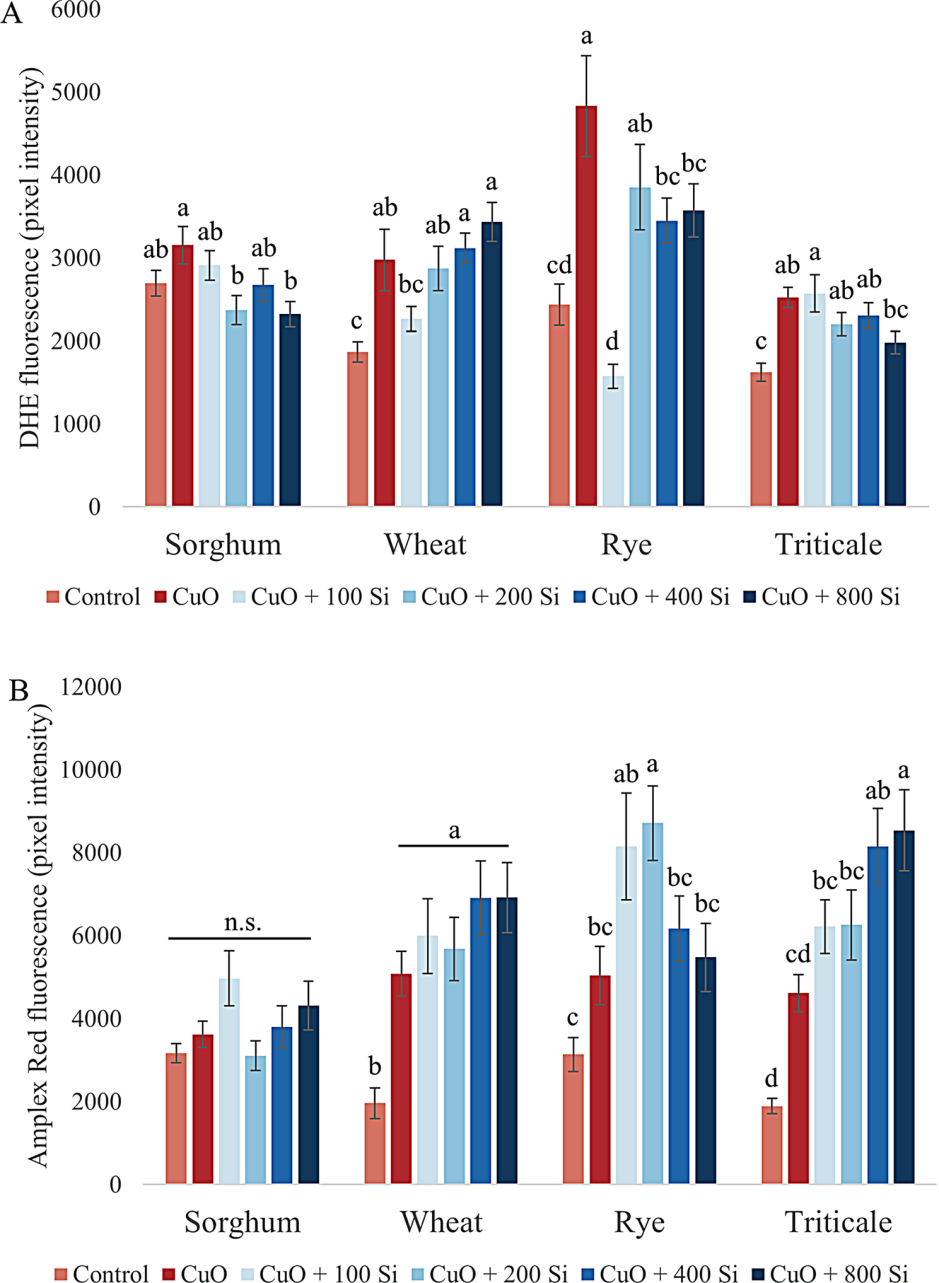
Changes in the levels of superoxide anion (**A**) and hydrogen peroxide (**B**) in sorghum, wheat, rye and triticale root tips under CuO nanoparticle stress and SiO_2_ nanoparticle pretreatment. The results are presented as the means ± standard errors (different letters indicate significant differences according to Duncan’s test; *P* ≤ 0.05; n.s., no significant change)

Disruption of plant growth is frequently accompanied by an imbalance in the homeostasis of reactive molecules. In the context of root growth responses, a change in the dynamics of reactive signal molecules was also observed in our experimental system. Among the most significant components of this signaling system, we initially examined the superoxide anion (O_2_˙^−^), which is the earliest ROS formed within the cell and thus plays a pivotal role in the development of oxidative stress (Fig. 2A).

In sorghum root tips, CuO treatment resulted in a slight increase in the amount of O_2_˙^−^, which was significantly reduced to below control levels by SiO_2_ NP treatments at 200 and 800 mg/L (*P* = 0.017 and 0.014, respectively). In contrast, the 100 and 400 mg/L SiO_2_ NP pretreatments resulted in similar amounts of O_2_˙^−^ as the control. In wheat, in contrast to the significantly increased O_2_˙^−^ levels induced by CuO NPs, pretreatment with 100 mg/L SiO_2_ NPs resulted in a notable difference (*P* = 0.056), with SiO_2_ NP treatment of seeds significantly reducing O_2_˙^−^ accumulation to levels that were only marginally different from those of the control. Conversely, SiO_2_ NP pretreatment at 200 mg/L did not affect the quantity of O_2_˙^−^, whereas at higher concentrations, it resulted in a slight increase in the quantity of O_2_˙^−^ in comparison with that in the treatment with only CuO NPs. The CuO NP treatment resulted in a notable increase in the concentration of O_2_˙^−^ in the rye. In contrast to CuO NP stress, SiO_2_ NP pretreatment at a concentration of 100 mg/L had the most pronounced effect (*P* < 0.001), with a significant reduction in O_2_˙^−^ levels in the meristematic zones of the roots, which were lower than those observed in the control root tips. The remaining three SiO_2_ NP pretreatments also resulted in a reduction in O_2_˙^−^ levels compared with those induced by the CuO NP treatment, albeit to a lesser extent. In triticale, the plants that received 800 mg/L SiO_2_ pretreatment presented the most pronounced difference in their CuO NP-induced response (*P* = 0.061). Conversely, the lowest treatment of 100 mg/L SiO_2_ NPs resulted in a slight increase in O_2_˙^−^ in the root tips.

Among the various reactive oxygen species, hydrogen peroxide (H_2_O_2_) is relatively less reactive; however, it can be toxic at elevated concentrations (Fig. 2B). In sorghum root tips, while CuO NP treatment did not result in an increase in H_2_O_2_ levels, 100 and 800 mg/L SiO_2_ NP pretreatments led to a slight increase in H_2_O_2_ levels. In wheat, CuO NP treatment resulted in a significant increase in H_2_O_2_ levels (*P* = 0.005), which were further elevated to a slight and nonsignificant extent by SiO_2_ NP pretreatment. In rye, H_2_O_2_ levels were significantly elevated at 200 mg/L (*P* = 0.02), and in triticale, H_2_O_2_ levels were significantly elevated at 400 and 800 mg/L (*P* = 0.008 and 0.005, respectively) compared with those in the CuO NP treatment in response to SiO_2_ NP pretreatment.

Heavy metals, as stressors, can disrupt the equilibrium of reactive oxygen species. Previous research has addressed the potential application of SiO_2_ to alleviate heavy metal stress, but the experimental systems employed in these studies differed from those presented here. Si seed treatment can mitigate the effects of Cu stress in wheat seedlings by reducing oxidative damage and decreasing Cu concentrations in plant tissues. The application of Si has been demonstrated to increase the activity of antioxidant enzymes, including peroxidase, catalase and superoxide dismutase [81]. In a study conducted by Tripathi et al. [82], Si was shown to reduce oxidative stress in pea plants under chromium stress.

Importantly, although the impact of SiO_2_ treatment on ROF metabolism has been demonstrated, there is a significant knowledge gap regarding its effects on RNF homeostasis.

**Fig. 3:**
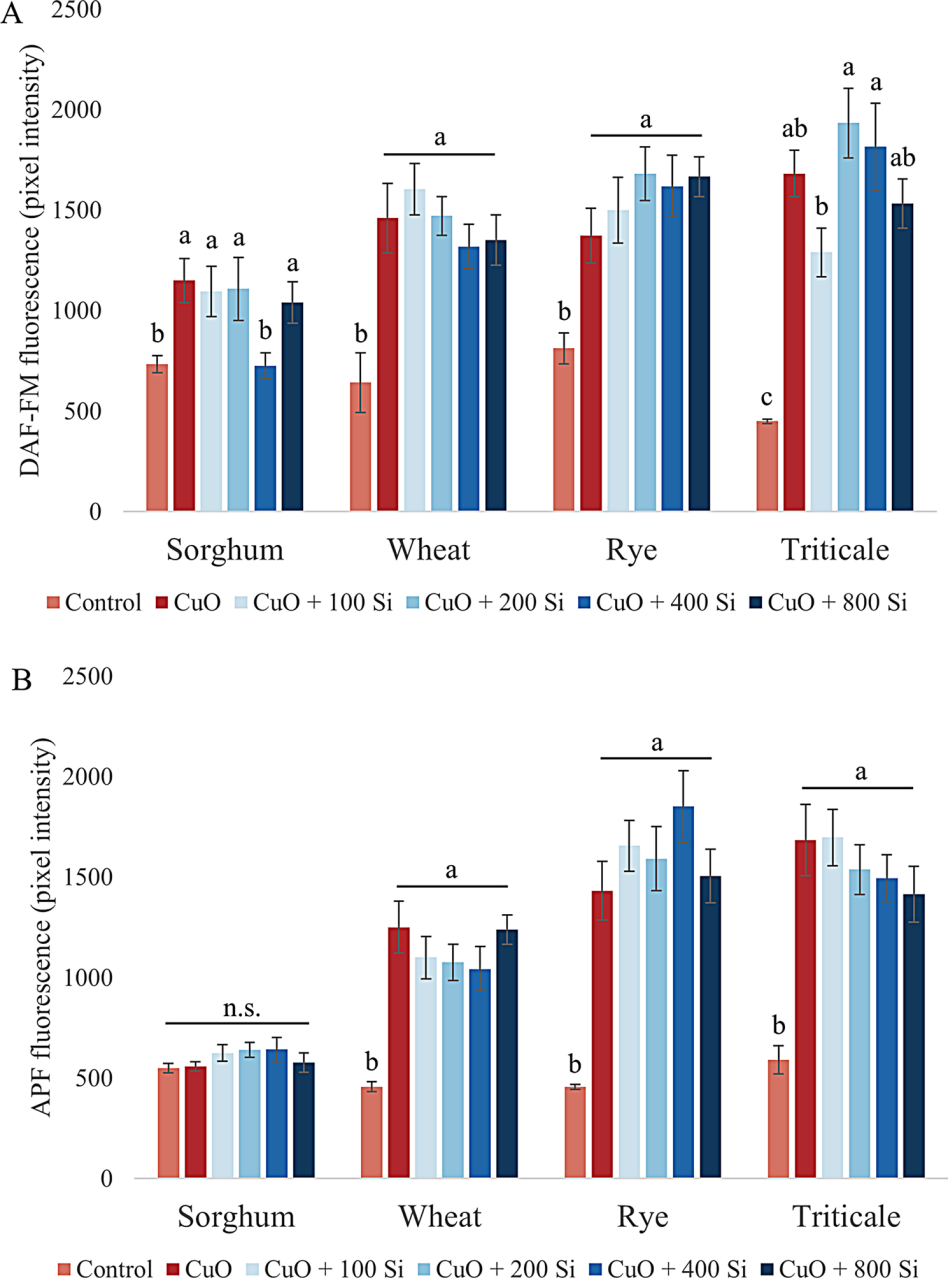
Changes in the levels of nitric oxide (**A**) and peroxynitrite (**B**) in sorghum, wheat, rye and triticale root tips under CuO nanoparticle stress and SiO_2_ nanoparticle pretreatment. The results are presented as the means ± standard errors (different letters indicate significant differences according to Duncan’s test; *P* ≤ 0.05; n.s., no significant change)

Nitric oxide (NO) is an important signal transduction molecule and the best-characterized reactive form of nitrogen, which plays a role in a number of biological processes (Fig. 3A). The levels of NO present in the root tips of the sorghum plants were significantly elevated as a consequence of the application of CuO NPs. The effect of SiO_2_ NP pretreatment at 100, 200 and 800 mg/L was only slight, whereas the 400 mg/L treatment resulted in a significant reduction (*P* = 0.009). In the wheat root tips, CuO NP-induced high NO levels slightly increased with the lowest concentration of SiO_2_ NPs, whereas higher concentrations tended to decrease NO levels in the root tips. In the case of rye, the treatments merely served to augment the accumulation of NO that had been induced by CuO NPs. In the triticale root tips, the lowest SiO_2_ NP core pretreatment resulted in a notable reduction in the amount of accumulated NO, whereas the 200 and 400 mg/L pretreatments resulted in a slight increase.

An illustrative example of the close relationship between ROS and RNS is the reaction of NO and O_2_˙^−^ to form peroxynitrite (ONOO^−^) (Fig. 3B). ONOO^−^ is capable of reacting with macromolecules, resulting in protein tyrosine nitration and subsequent loss of function and rapid degradation [83]. The ONOO^−^ content in sorghum root tips was not affected by CuO NP stress, and only a slight accumulation was detected in response to SiO_2_ NP pretreatment. The significantly elevated ONOO^−^ levels observed in wheat root nodules subjected to CuO NP stress gradually decreased, although not significantly, in response to SiO_2_ NP treatment. However, at a concentration of 800 mg/L, the ONOO^−^ levels approached those induced by CuO NPs. Furthermore, additional ONOO^−^ accumulation was observed in the rye root tips, in addition to the significant accumulation induced by CuO NPs as a result of SiO_2_ NP pretreatment. Conversely, in triticale root tips, SiO_2_ NP pretreatment resulted in a nonsignificant reduction in ONOO^−^ levels, which nevertheless exhibited a concentration-dependent decline.

**Fig. 4:**
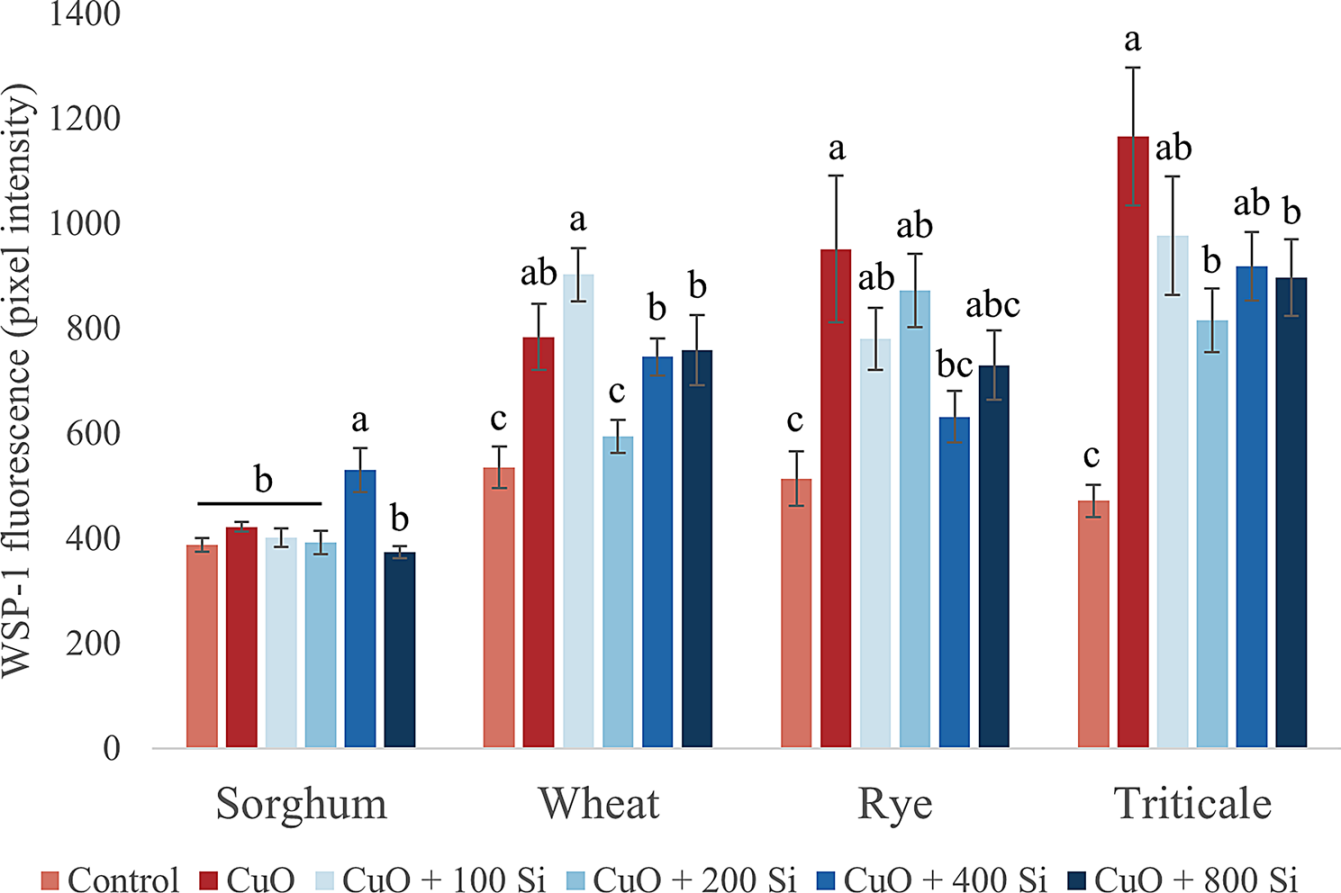
Changes in the levels of hydrogen sulfide in sorghum, wheat, rye and triticale root tips under CuO nanoparticle stress and SiO_2_ nanoparticle pretreatment. The results are presented as the means ± standard errors (different letters indicate significant differences according to Duncan’s test; *P* ≤ 0.05; n.s., no significant change)

H_2_S is a gaseous signaling molecule that induces lateral root formation, plays an important role in seed germination and fruit ripening, is involved in stress response reactions, and can also form free radicals [84] (Fig. 4). In the meristematic zone of sorghum, treatment with CuO NPs did not result in any change in H_2_S levels. However, pretreatment with SiO_2_ NPs at a concentration of 400 mg/L led to a significant increase in H_2_S levels (*P* = 0.032). On the other hand, wheat and triticale presented comparable responses. CuO NP stress resulted in significant accumulation of H_2_S in the root tips of both species, whereas the 200 mg/L SiO_2_ NP pretreatment had a significant effect on reducing H_2_S accumulation (wheat: *P* = 0.033; triticale: *P* = 0.01). Additionally, CuO NP treatment resulted in a significant increase in H_2_S levels in rye, which is indicative of stress. Conversely, the 400 mg/L SiO_2_ NP treatment led to a significant reduction in H_2_S levels (*P* = 0.015).

### Examining further changes

Plants have evolved a variety of defensive mechanisms to protect themselves against the toxic effects of heavy metals. One such mechanism is the modification of cell walls. Such modifications may include an increase in cell wall thickness or alterations in chemical composition [85]. In the case of dwarf banana, the application of silicon was observed to assist in maintaining the sodium/potassium balance and to reduce damage to the cell walls, as evidenced by Mahmoud et al. [86]. Furthermore, the use of a silicon suspension was demonstrated to increase the uptake and transport of silicon nanoparticles in fenugreek while also increasing the degree of cell wall lignification, as documented by Nazaralian et al. [87].

**Fig. 5:**
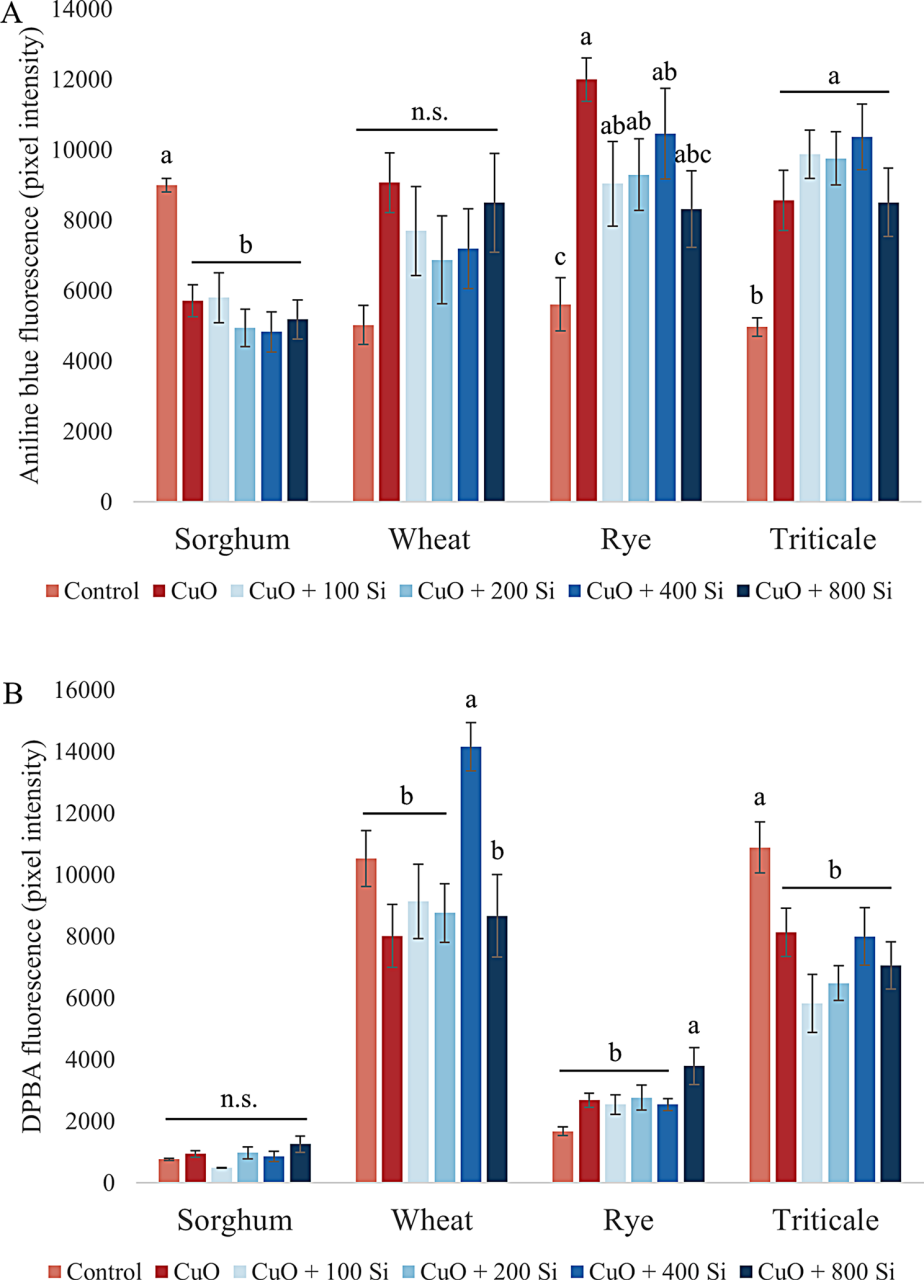
Changes in the callose content (**A**) and quercetin levels (**B**) in sorghum, wheat, rye and triticale root tips under CuO nanoparticle stress and SiO_2_ nanoparticle pretreatment. The results are presented as the means ± standard errors (different letters indicate significant differences according to Duncan’s test; *P* ≤ 0.05; n.s., no significant change)

Like cellulose, callose is a polysaccharide with structural and protective functions in plant cell walls. It is postulated that callose represents the primary mechanical defense system of plants against environmental stresses [88] (Fig. 5A). The quantity of callose present in the root tips of sorghum plants exhibiting relatively high susceptibility was diminished by CuO NP stress, with a further reduction observed in plants subjected to higher concentrations of SiO_2_ NP pretreatment. Conversely, in the more tolerant species, elevated levels of callose deposition were observed in response to CuO NP stress. The callose content of the root tips of wheat and rye tended to decrease in response to SiO_2_ NP pretreatment. Conversely, in triticale, the quantity of callose within the cell walls of the root tip persisted in its upward trajectory.

Quercetin is a flavonoid that plays a role in several plant processes, including seed germination, growth and development. It is a potent antioxidant, rendering it effective against a number of biotic and abiotic stresses [89] (Fig. 5B). The concentration of quercetin in sorghum root tips was also lower under control conditions than in the other three species studied. The application of CuO NP stress resulted in a slight increase in the quantity of quercetin present in the root tips. However, this increase was only marginally enhanced by SiO_2_ NP pretreatment at elevated concentrations. Conversely, the lowest concentration of SiO_2_ NPs led to a notable reduction in the amount of quercetin observed in the root tips. In wheat, the application of CuO NPs resulted in a slight reduction in the quercetin content of the root tips. Among the SiO_2_ NP pretreatments, only a concentration of 400 mg/L resulted in significant accumulation (*P* < 0.001). The quercetin content of the rye root tips was lower than that of the wheat and triticale root tips. The application of CuO NPs slightly increased the quantity of quercetin present in the root tips. However, this effect was significantly enhanced by the highest concentration of SiO_2_ NPs used as a pretreatment (*P* = 0.035). In triticale, the quantity of quercetin present in the root tip was observed to decline significantly as a consequence of CuO NP stress.

### Investigation of changes in protein tyrosine nitration

**Fig. 6:**
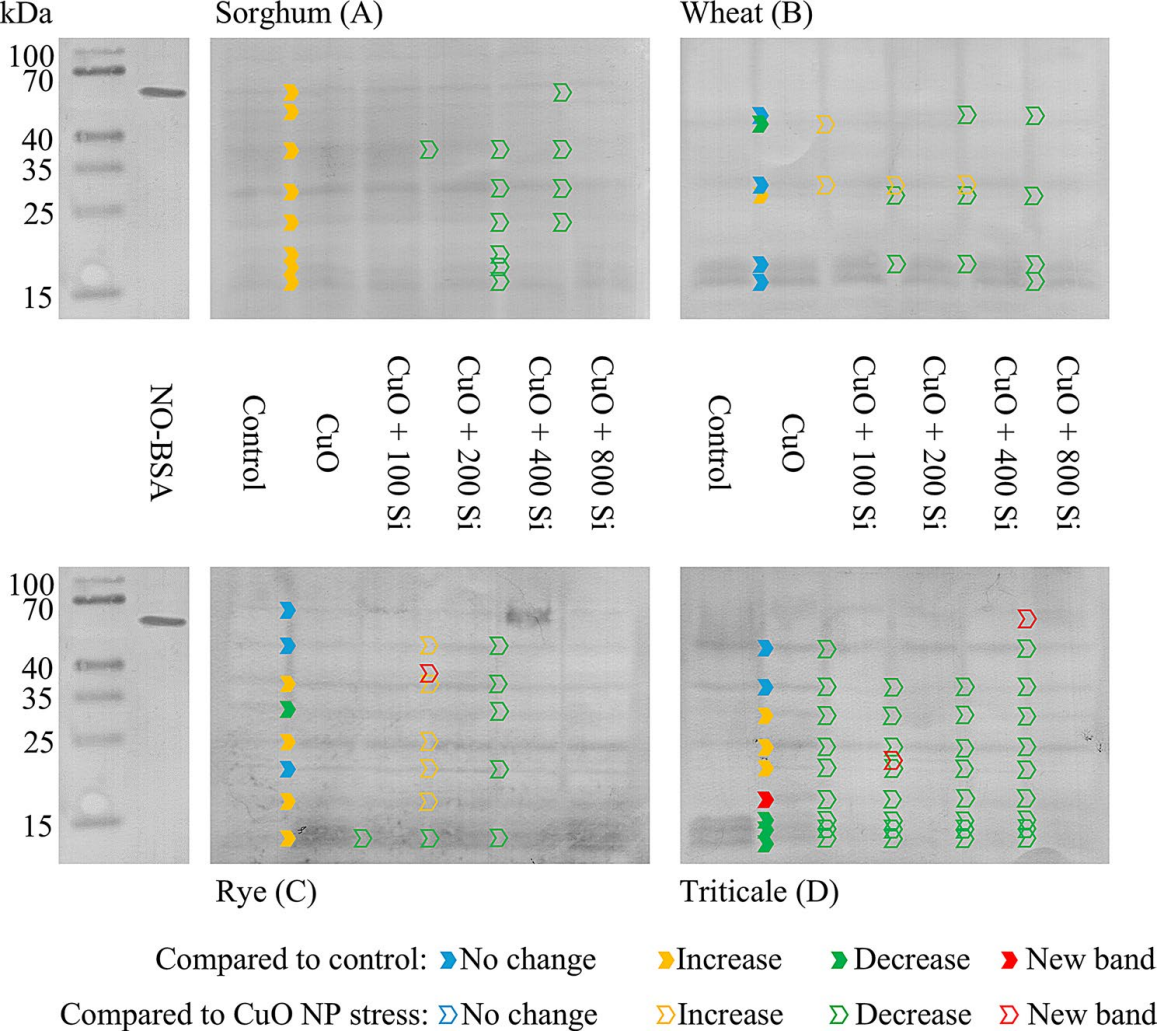
Representative immunoblot showing protein tyrosine nitration in sorghum (**A**), wheat (**B**), rye (**C**) and triticale (**D**) roots under control conditions and after pretreatment with different amounts of SiO_2_ NPs and CuO NP stress. The blue arrows indicate unchanged nitrated protein bands, the red arrows indicate new nitrated bands, the yellow arrows indicate increased nitrated protein bands, and the green arrows indicate decreased nitrated protein bands (NO-BSA: nitrated bovine serum albumin, used as a positive control)

Protein nitration was detected via Western blot analysis as a marker of nitro-oxidative stress in the root system of monocots (Fig. 6).

As previously reported, bands indicative of nitration were also detected in control plants, indicating that the process of protein nitration occurs under stress-free physiological conditions [53, 73]. In sorghum roots, CuO NPs increased nitration, whereas SiO_2_ NPs at concentrations of 400 and 800 mg/L decreased nitration of several protein bands in comparison with CuO NP stress (Fig. 6A). In wheat, fewer nitrated protein bands were observed under control conditions, with differing responses in terms of intensity to CuO NP stress. Compared with CuO NP stress, the application of SiO_2_ NPs resulted in the emergence of several bands with increased intensity (40, 35 kDa); however, in the majority of cases, the utilization of SiO_2_ NPs led to a reduction in nitration (Fig. 6B). In rye roots, CuO NP stress resulted in increased nitration of several protein bands. Additionally, a new band appeared in the 40 kDa size range for the 200 mg/L SiO_2_ NP pretreatment, as did five enhancing bands in the range of 40 − 15 kDa relative to CuO NP stress. In contrast, the 400 mg/L SiO_2_ NP treatment resulted in a uniform reduction in the nitration signal (Fig. 6C). In triticale roots, with the exception of the 25 kDa band of the 200 mg/L pretreated sample and the 70 kDa band of the 800 mg/L sample, the nitration signal was reduced by SiO_2_ NP pretreatment in comparison with the changes induced by CuO NP stress (Fig. 6D).

Protein tyrosine nitration plays a role in a number of stress-related processes [90]. In previous studies examining the effects of heavy metals on protein nitration, nickel was observed to increase this process to a lesser extent in *Brassica juncea* than in *Arabidopsis thaliana* [91]. An earlier unpublished result demonstrated that ionic copper stress did not induce protein tyrosine nitration in *Brassica juncea* or *Brassica napus* plants [92]. This differs from the changes in the extent of CuO NP-induced protein tyrosine nitration observed in this experimental system, indicating that the occurrence and extent of this process may be influenced by both the plant species under study and the form of copper treatment.

## Discussion

In a previous study, consistent mechanisms were identified that contributed to the 50% inhibition of root length growth. In the case of the inhibition observed in the relatively sensitive sorghum, a smaller concentration of CuO nanoparticles was needed. This was accompanied by a lack of significant alterations in the levels of reactive forms in the root tips; however, strong protein tyrosine nitration was detected. In contrast, a markedly greater quantity of CuO NPs was necessary to impede the growth of wheat, rye, and triticale, which demonstrated relatively high tolerance. While the levels of reactive forms were markedly elevated in the background, nitration did not exhibit a similar increase [53].

CuO nanoparticles have been shown to inhibit plant growth primarily through the generation of ROS. The presence of copper ions (Cu^2+^) released from the dissolution of CuO nanoparticles can induce oxidative stress by participating in Fenton-like reactions, leading to the overproduction of ROS such as O_2_˙^−^, hydroxyl radicals, and H_2_O_2_. This excessive ROS generation can damage cellular components such as lipids, proteins, and DNA; disrupt cellular signaling pathways; and ultimately inhibit plant growth and development [93–96].

**Fig. 7:**
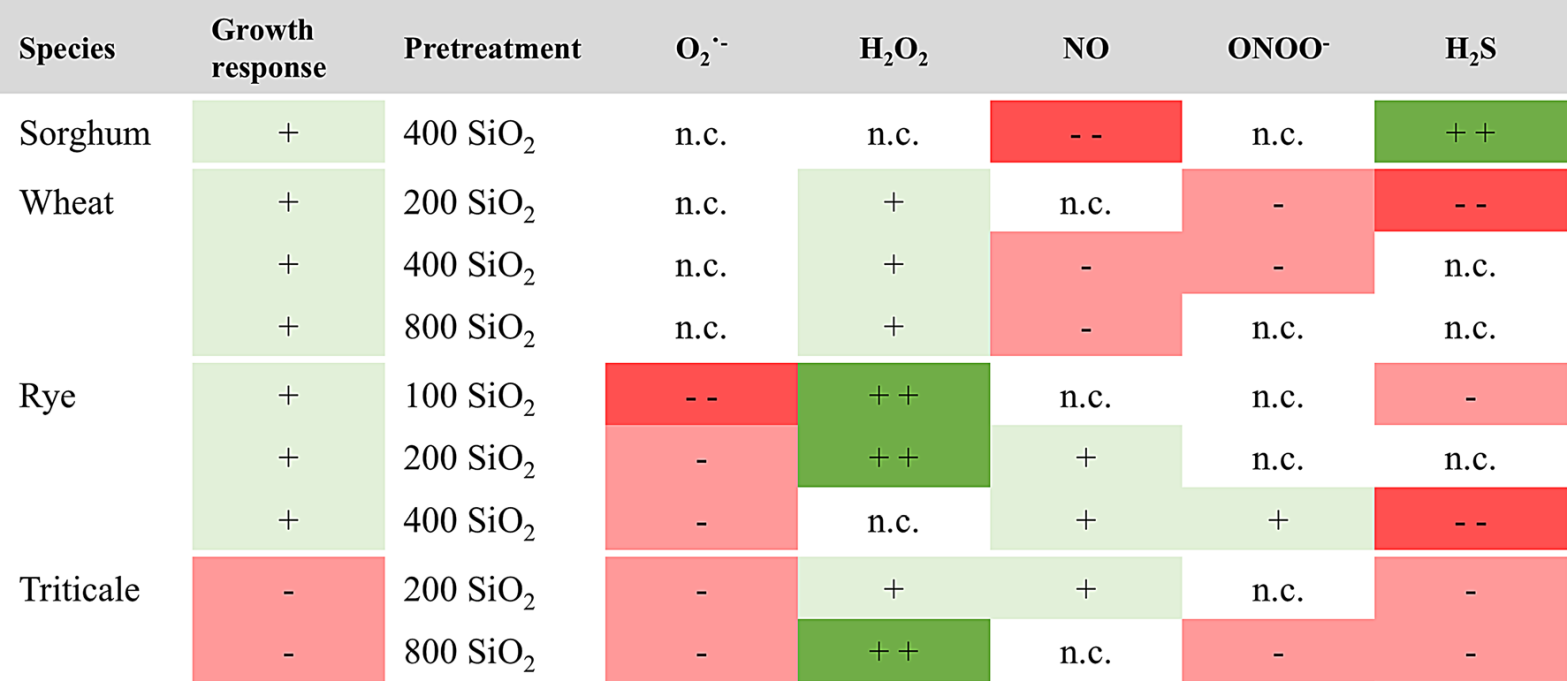
Effects of SiO_2_ nanoparticle pretreatment of monocot seedlings on their subsequent CuO-induced stress responses. Different shades of green represent an increasing trend, different shades of red represent a decreasing trend, and n.c. represents no significant change

This study provides valuable insights into the species-specific effects of SiO_2_ NP pretreatment on mitigating CuO NP-induced stress in monocot crops. By systematically examining root growth responses and nitro-oxidative stress markers, we observed that SiO_2_ NP pretreatment can produce both alleviating and exacerbating effects depending on the species, concentration, and molecular context (Fig. 7.).

Our findings revealed distinct species-specific responses to SiO_2_ NP pretreatment. In sorghum, SiO_2_ NP pretreatment effectively alleviated CuO NP-induced root growth inhibition, accompanied by reduced NO levels and elevated H_2_S levels. This suggests a shift in nitro-oxidative balance, likely reflecting enhanced antioxidant defenses in this relatively sensitive species. Similar mitigation effects were observed in wheat and rye; however, the underlying molecular changes differed. Wheat presented a general reduction in RNS and increased H_2_O_2_ levels, indicative of a stress-tolerant response linked to SOD activity. In rye, reduced O_2_˙⁻ levels with increased H_2_O_2_ suggest efficient detoxification of ROS.

In contrast, triticale displayed exacerbated stress under SiO_2_ NP pretreatment, with further inhibition of root growth observed at specific concentrations. This was coupled with inconsistent changes in RNS and ROS, indicating dysregulated signaling pathways. The observed inhibitory effects at higher SiO_2_ NP concentrations may reflect a shift from beneficial priming effects to toxic nanoparticle accumulation, disrupting cellular processes and leading to growth inhibition. These species-specific responses underscore the need for tailored nanoparticle-based interventions that consider crop-specific physiological and molecular traits.

Protein tyrosine nitration, a marker of nitro-oxidative stress, was significantly modulated by SiO_2_ NP pretreatment. CuO NPs uniformly increased the nitration intensity across species, which is consistent with prior studies [53]. SiO_2_ NP pretreatment, however, reduced nitration in most cases, suggesting the mitigation of nitro-oxidative stress. Notably, new nitrated protein bands appeared in rye and triticale, potentially indicative of unique acclimation or stress responses in these species [97].

The present study demonstrated that the pretreatment of seeds with SiO_2_ NPs was able to reduce CuO NP-induced growth inhibition in several cases. However, the underlying changes in the homeostasis of reactive forms are not uniform across species. While CuO NPs function as stressors, inducing a uniform nitro-oxidative response in wheat, rye, and triticale roots, the positive effect of SiO_2_ NP pretreatment under CuO stress can be attributed to a different species-dependent nitro-oxidative response. The contrasting effects of CuO and SiO_2_ NPs on monocots are clearly discernible. SiO_2_ NPs elicited a complex and nuanced response, with the stabilization of reactive molecule homeostasis and a decrease in nitration intensity observed in the majority of cases. This selective mitigation by SiO_2_ NPs indicates that their interaction with plant signaling pathways enables acclimation to CuO-induced stress through various responses in reactive signaling, which differs significantly from the generalized stress caused by CuO NPs alone.

The available literature does not directly address the combined effects of silica nanoparticles and metal oxide nanoparticles on plants and provides insights into only the individual effects of these NPs. Several previous studies have investigated the beneficial effects of silica nanoparticles on plant growth and development, including under abiotic stress conditions. The mechanisms by which silicon confers stress tolerance involve both passive and active uptake processes, as well as the regulation of various signaling pathways (reviewed by 98, 99). Silica nanoparticles can increase the activity of antioxidant enzymes in barley plants under water stress, improving their growth and yield [100], and they can upregulate the expression of genes involved in the antioxidant defense system in maize plants under drought stress [101]. It is also important to consider the potential phytotoxic effects of silica NPs, as a previous study has demonstrated that at higher concentrations, the uptake of silica NPs can lead to oxidative stress and damage in *Arabidopsis thaliana* [102]. At relatively high concentrations, SiO_2_ NPs can accumulate excessively in root tissues, disrupting cellular homeostasis by causing physical blockage of apoplastic or symplastic transport pathways [103]. This interference may impair the uptake and transport of nutrients and water, adversely affecting plant growth. Furthermore, the effects of SiO_2_ NPs are dose dependent (i.e., 104), with beneficial effects observed at moderate levels. However, beyond certain thresholds, their interactions with cellular components can transition from protective to toxic, highlighting the importance of optimizing nanoparticle concentrations for safe and effective applications.

The observed ability of SiO_2_ NPs to modulate nitro-oxidative balance in sorghum, wheat, and rye highlights their potential utility in enhancing crop resilience under CuO nanoparticle stress. However, the exacerbated stress in triticale points to the necessity of tailoring nanoparticle-based interventions to specific crop species.

The complex and multifaceted interactions between nanomaterials and plants, involving physicochemical, physiological, biochemical, and genetic factors, contribute to the differences in plant responses to nanomaterials. The specific properties of nanomaterials, such as their size, shape, surface chemistry, and composition, can influence their interactions with and effects on different plant species [105–107]. Moreover, the ability of nanomaterials to be taken up and translocated within different plant species varies, depending on factors such as root architecture, cell wall permeability, and specific transport mechanisms [108]. Furthermore, plants have evolved diverse mechanisms to respond to abiotic and biotic stresses, including the oxidative stress induced by nanomaterials. The efficiency of these defense systems can vary among plant species and cultivars (109–110). Exposure to nanomaterials can induce changes in gene expression, DNA methylation, and other epigenetic modifications, which can lead to diverse phenotypic responses among plant species and cultivars [106, 108]. Additionally, the effects of nanomaterials on plants can be influenced by various environmental factors, such as soil properties, water availability, temperature, and light, which can vary across different growth conditions (111–112).

Our findings address a critical knowledge gap by demonstrating that SiO_2_ NP pretreatment does not yield uniform mitigation effects across all monocots but instead produces species-specific responses. This nuanced understanding challenges the existing paradigm of generalized nanoparticle applications and highlights the need for crop-specific strategies. Furthermore, the observed differences in nitro-oxidative stress responses, including alterations in NO, H_2_S, O_2_˙⁻, and H_2_O_2_ levels, underscore the importance of tailoring nanoparticle applications to exploit these signaling pathways effectively. To our knowledge, this is one of the first comparative studies to explore both the mitigation and exacerbation effects of SiO_2_ NPs under CuO NP stress, providing valuable insights into the molecular mechanisms involved.

Although this study offers valuable insights into the potential of silica nanoparticles to mitigate CuO NP-induced stress in monocots, it is also important to acknowledge their limitations. The experiments were conducted in a controlled in vitro semihydroponic system, which may not fully replicate the complex environmental interactions encountered in field conditions. Additionally, the species-specific effects observed suggest that further research is needed to gain insight into the molecular mechanisms underlying these responses and to explore the long-term implications of nanoparticle use in diverse crop systems. Future studies integrating field trials and investigating broader ecological impacts will be crucial for translating these findings into sustainable agricultural practices.

## Conclusions

The objective of this study was to examine the impact of SiO_2_ NP pretreatment on CuO NP-induced stress responses in agriculturally significant monocot plants. To further investigate the potential of SiO_2_ NPs to mitigate the effects observed, this study builds on previous work conducted by Kacziba et al. [53]. The results demonstrated that SiO_2_ NP pretreatment effectively mitigated CuO NP-induced root growth inhibition in sorghum, wheat, and rye but unexpectedly intensified growth inhibition in triticale.

The species-specific effects of SiO_2_ NPs were accompanied by distinct changes in the homeostasis of reactive molecules. Pretreatment with SiO_2_ NPs resulted in a reduction in NO and an increase in H_2_S in sorghum, an increase in H_2_O_2_ in wheat, and a decrease in O_2_˙^−^ in rye. These findings indicate that SiO_2_ NPs engage in different nitro-oxidative pathways, which tailor reactive signaling responses to counteract CuO NP stress in a species-dependent manner.

By addressing the differential effects of SiO_2_ NP pretreatment on CuO NP-induced stress in sorghum, wheat, rye, and triticale, this study fills a critical knowledge gap in the field of plant stress physiology. The species-specific modulation of nitro-oxidative signaling pathways, coupled with the reduction in protein tyrosine nitration, highlights the potential of SiO_2_ NPs as tools for targeted stress mitigation. These findings lay a foundation for the application of nanotechnology in precision agriculture, offering promising strategies to increase crop resilience to abiotic stressors. Future research should focus on validating these results under field conditions and expanding the scope to other crops and nanoparticle types to develop comprehensive, sustainable agricultural solutions.

## Electronic supplementary material

Below is the link to the electronic supplementary material.

Supplementary Material 1

## Data Availability

All the data generated or analyzed during the course of this study are included in the graphs presented in the published article.
