# Supplementary material for: Species-specific modulation of nitro-oxidative stress and root growth in monocots by silica nanoparticle pretreatment under copper oxide nanoparticle stress
Source: BMC Plant Biol. 2025 Feb 13;25:188. doi: 10.1186/s12870-025-06193-7 (PMC11823027; doi:10.1186/s12870-025-06193-7)
Supplement: Supplementary file 1 — Supplementary Material 1 [file 12870_2025_6193_MOESM1_ESM.docx]

**Supplementary materials**

**1. Root growth responses to SiO_2_ NP priming**


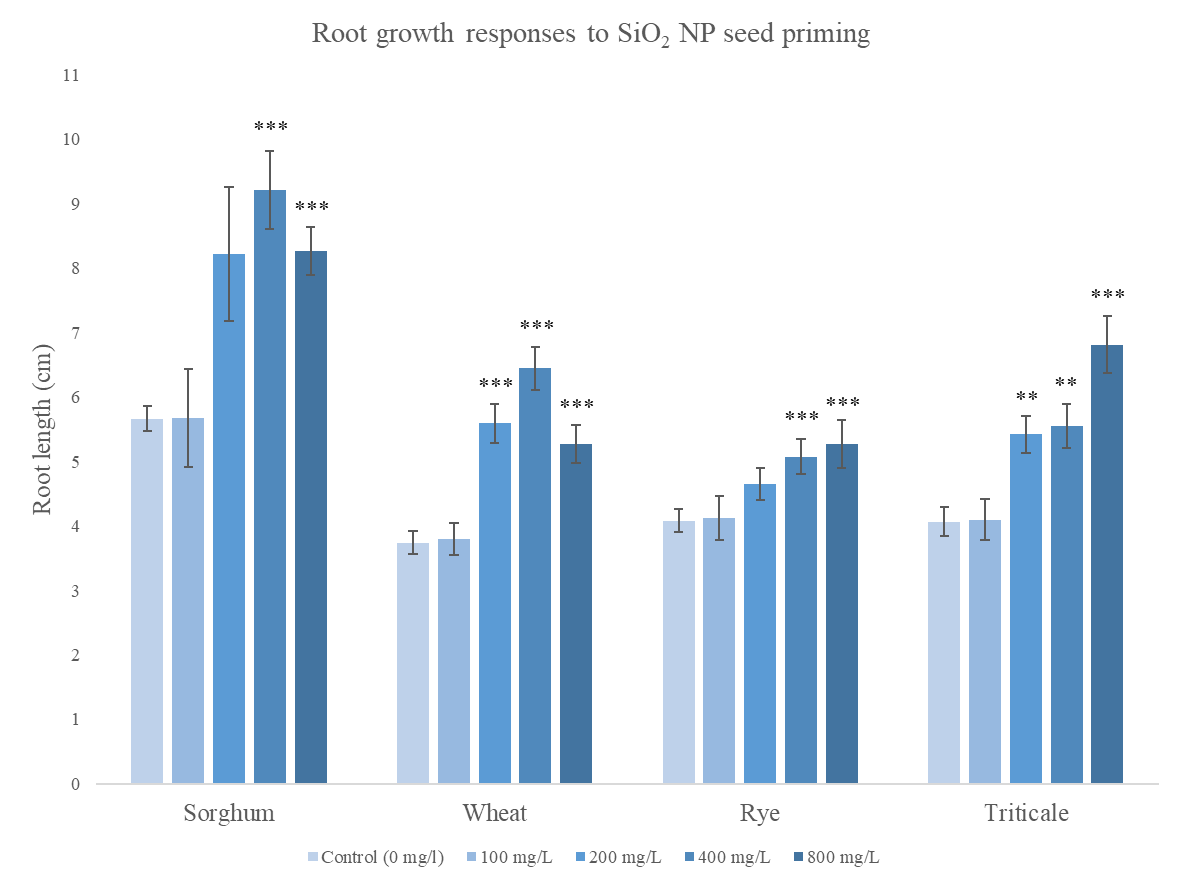


Figure S1. Root growth responses to seed priming with different concentrations of SiO_2_ nanoparticles. Asterisks indicate a significant difference from the control by Student's t-test (*P≤0.05, **P≤0.01, ***P≤0,001).

**2. Characterization of SiO_2_ NPs**

**Methods**

Transmission electron microscopy (TEM) measurements were applied to characterize the morphology and size of SiO_2_ NPs using a FEI Tecnai G2 20× microscope ((FEI Corporate Headquarters, Hillsboro, OR, USA) at an acceleration voltage of 200 kV. The crystal structure of SiO_2_ nanoparticles were verified by X-ray powder diffraction (XRD). The scans were recorded with a Rigaku MiniFlex II powder diffractometer (Rigaku Corporation, Tokyo, Japan) using Cu Kα radiation and a scanning rate of 2° min−1 in the 5°–80° 2θ range.

**Results**

**Chemical properties of SiO_2_ NPs**

The obtained SiO_2_ nanoparticles were analyzed by TEM, which indicated that the particles are generally uniform and aggregated (Figure S1A). Image analysis revealed that the average size of the particles proved to be between 10-20 nm. Figure S1B shows the X-ray powder diffraction pattern of SiO_2_ nanoparticles. Only one broadened peak, with a Bragg angle of 2θ = 22.8º, was recorded, which corresponds to the amorphous phase according to the literature (1). The silica nanoparticles showed colloidal stability with a highly negative surface charge (-39.1 mV).


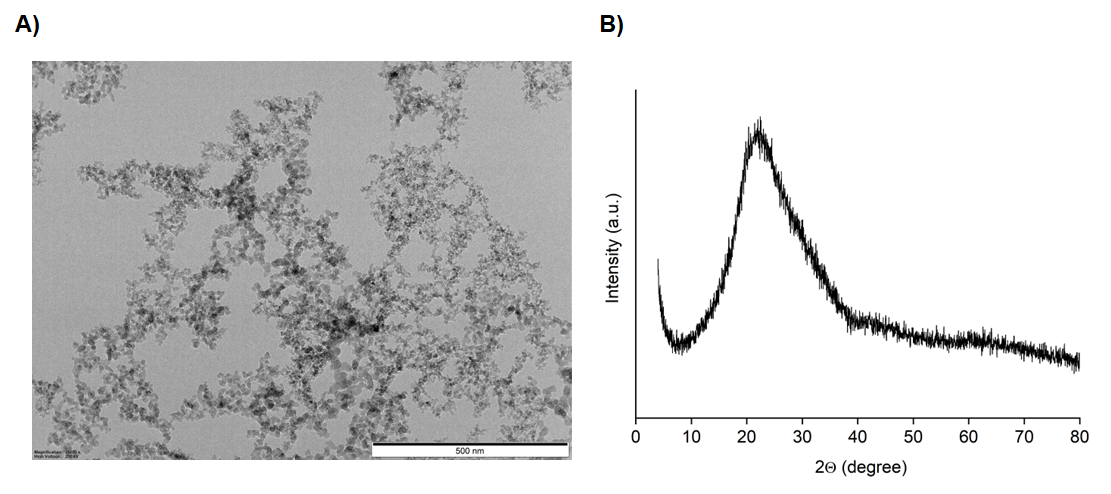


**Figure S2. Chemical characterization of silica nanoparticles.** Transmission electron microscopic (TEM) image (A), and X-ray diffractogram (XRD) of the synthesized SiO_2_ particles (B).

1. Kim, K. M., Kim, H. M., Lee, W. J., Lee, C. W., Kim, T. I., Lee, J. K., ... & Oh, J. M. (2014). Surface treatment of silica nanoparticles for stable and charge-controlled colloidal silica. International journal of nanomedicine, 9(sup2), 29-40.
2. Martínez, J. R., Palomares-Sánchez, S., Ortega-Zarzosa, G., Ruiz, F., & Chumakov, Y. (2006). Rietveld refinement of amorphous SiO2 prepared via sol–gel method. Materials letters, 60(29-30), 3526-3529.
